# Case report: Gastric carcinoma with SMARCA4 deficient: two cases report and literature review

**DOI:** 10.3389/fonc.2024.1297140

**Published:** 2024-02-06

**Authors:** Zeyang Lin, Qian Li, Yujie He, Shujing Guo, Yuhan Ye, Zhengjin Liu

**Affiliations:** ^1^ Department of Pathology, Zhongshan Hospital of Xiamen University, School of Medicine, Xiamen University, Xiamen, China; ^2^ Lianqian Street Community Health Service Center, First Affiliated Hospital of Xiamen University, Xiamen, China

**Keywords:** SMARCA4, gastric carcinoma, category, therapy, case

## Abstract

SMARCA4-deficient gastric carcinoma has been reported sporadically since 2016. Only 29 patients have been reported; nevertheless, it is aggressive and highly malignant with poor outcomes. It has an immunohistochemical phenotype showing loss of SMARCA4 expression and can be accompanied by codeletion of other switch/sucrose non-fermentable chromatin-remodeling complex subunits. Microscopically, it displays high-grade undifferentiated histological morphology with rhabdoid cell differentiation. Rarely does the tumor contain a purely or partly adenocarcinoma component. Here, we report two cases to demonstrate these unusual morphologies analyzed using morphological and immunohistochemical techniques. In addition, there is a lack of research on the classification of these morphologies. Therefore, our report will aid the diagnosis and classification of SMARCA4-deficient gastric carcinoma.

## 1 Introduction

Undifferentiated gastric carcinoma is a primary tumor without specific cytological or architectural types of differentiation (1). Expression of switch/sucrose non-fermentable (SWI/SNF) chromatin-remodeling complex subunits is reportedly deficient in some cases; these subunits include SMARCA4, SMARCA2, SMARCB1, and ARID1A. SMARCA4-deficient undifferentiated carcinomas (SD-UCs) are rare and were described first in 2016 by Agaimy et al. (2) The morphological features are solid, diffuse sheets of polygonal cells with pleomorphic giant cells. These cells have vesicular nuclei and a high degree of mitosis, which are poorly cohesive. A rhabdoid cell component is common and may be the predominant pattern. Because rhabdoid cells are a diagnostic clue, the terminology undifferentiated/rhabdoid carcinoma was proposed by Chang et al. (3) A few cases of SMARCA4-deficient undifferentiated gastric carcinoma were reported demonstrating glandular differentiation or encompassing adenocarcinoma. According to the fifth World Health Organization (WHO) classification of the digestive system, undifferentiated gastric carcinoma is a malignant epithelial tumor composed of anaplastic cells with no specific cytologic or architectural differentiation, including glandular, squamous, neuroendocrine, and sarcomatoid differentiation (1). In this context, “dedifferentiated carcinoma” was recommended for cases with adenocarcinoma components (3). There are also SMARCA4-deficient adenocarcinomas (SD-ADs), as we and Huang described (4). SMARCA4-deficient gastric carcinoma can be classified as SD-UC, where all components are undifferentiated, except the adenocarcinoma portion, according to the WHO. SMARCA4-deficient dedifferentiated carcinoma (SD-DC) is the term used for tumors with adenocarcinoma components. SD-AD is used for cases comprising purely adenocarcinoma components. These tumors are then categorized as well, moderately, or poorly differentiated based on the percentage of glandular components. Although, the latter two are not included in WHO classification.

The three subtypes have different immunophenotypes; panCK, SMARCA2, and E-cadherin are positive in adenocarcinoma areas; the opposite is true in undifferentiated areas. Attention should be paid to discriminating among the three subtypes to classify these unusual tumors.

## 2 Methods

Cases from Zhongshan Hospital of Xiamen University were reviewed, and the diagnosis of SD-DC and SMARCA4-deficient poorly differentiated adenocarcinoma was confirmed separately. All specimens were fixed with 3.7% neutral formaldehyde, dehydrated, paraffin-embedded, and cut into 4-μm serial sections. The sections were subjected to hematoxylin and eosin and immunohistochemical staining. The latter was performed using a two-step EnVision method. The primary antibodies included SMARCA4 (BRG1), SMARCB1 (INI-1), broad-spectrum cytokeratin (panCK), vimentin, cytokeratin (CK) 7, E-cadherin, CD34, CD56, synaptophysin (Syn), chromogranin A (CgA), spalt-like transcription factor 4 (SALL-4), Ki-67, p53, alpha-fetoprotein (AFP), and hepatocyte paraffin 1 (Hepa-1) and were purchased from Fuzhou Maixin Biotechnology Ltd. SMARCA2 (BRM) (Clone number ARC59944) was purchased from Abclonal Biotechnology, Ltd.

## 3 Case presentation

### 3.1 Case 1

**Figure 1:**
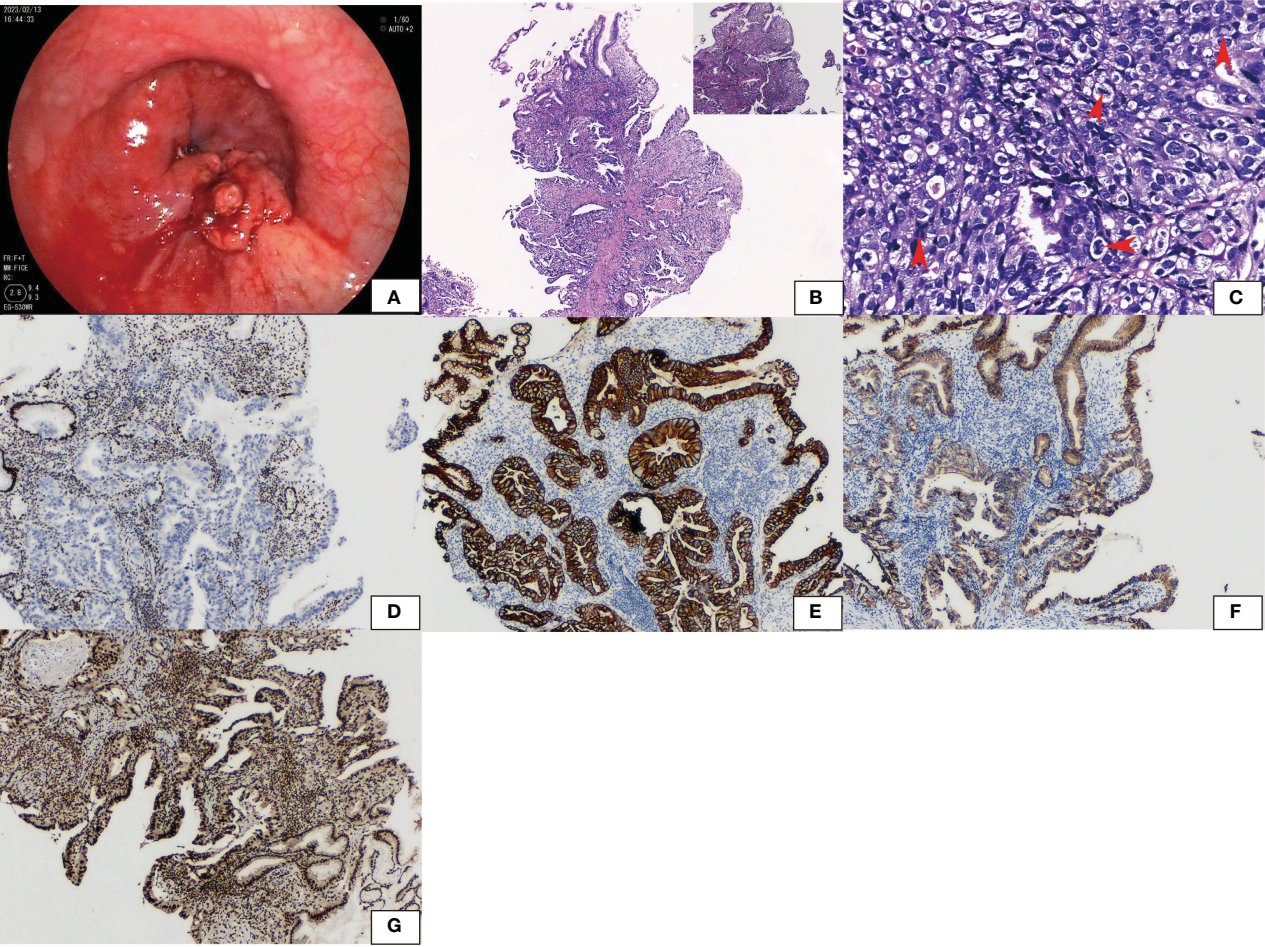
Gastroduodenoscopy showed **(A)** a cauliflower-like tumor with an ulcer in the cardia. **(B)** Hematoxylin and eosin staining showed glandular adenocarcinoma gradually transferred from the normal epithelium. High-grade intraepithelial neoplastic was seen with differentiated, moderately differentiated, and poorly differentiated adenocarcinoma. **(C)** Cells had large round to oval nuclei and coarse chromatin. The degree of mitosis was high (arrow). **(D)** Immunohistochemical staining revealed that SMARCA4 was lost in tumors while normal epithelium was retained. **(E)** PanCK, **(F)** E-cadherin, and **(G)** SMARCA2 were both positive.

A 65-year-old man presented to the Department of Gastroenterology, Zhongshan Hospital of Xiamen University, with a one-month history of indigestion after meals accompanied by pain in the right upper abdomen without any known inducement. Gastroduodenoscopy revealed a cauliflower-like mass with an ulcer in the cardia of the stomach (**Figure 1A**), and a biopsy was performed.

The pathological findings revealed a malignant tumor of epithelial origin. A glandular adenocarcinoma arose from the normal epithelium. High-grade intraepithelial neoplastic was present. There were features of differentiated, moderately differentiated, and poorly differentiated adenocarcinoma (**Figure 1B**). Cells had large round to oval nuclei and coarse chromatin. The mitosis index was 15/2 mm^2^ (**Figure 1C**). None of the tumor expressed SMARCA4 (**Figure 1D**). However, panCK (**Figure 1E**), CK7, SALL4, E-cadherin (**Figure 1F**), SMARCB1, and SMARCA2 (**Figure 1G**) were strongly positive. Synaptophysin was weakly positive. P53 was diffusely expressed. The tumor was negative for CD34, CgA, CD56, AFP, and Hepa-1. The tumor was finally diagnosed as SMARCA4-deficient poorly differentiated adenocarcinoma.

Computed tomography revealed liver and lymphatic metastases. After careful consideration, the patient and his family chose not to undergo further treatment. Clinical follow-up was available, and the patient was still alive more than 3 months from the date of diagnosis.

### 3.2 Case 2

A 75-year-old man presented with persistent hematochezia and melena associated with peripheral neuropathy and abdominal pain for over a month. Gastroduodenoscopy revealed a large mass occupying 50% of the antrum. Computed tomography revealed no lymphatic metastases. He was diagnosed with a malignant gastric tumor at another hospital. He was transferred to Zhongshan Hospital of Xiamen University and underwent gastrectomy.

**Figure 2:**
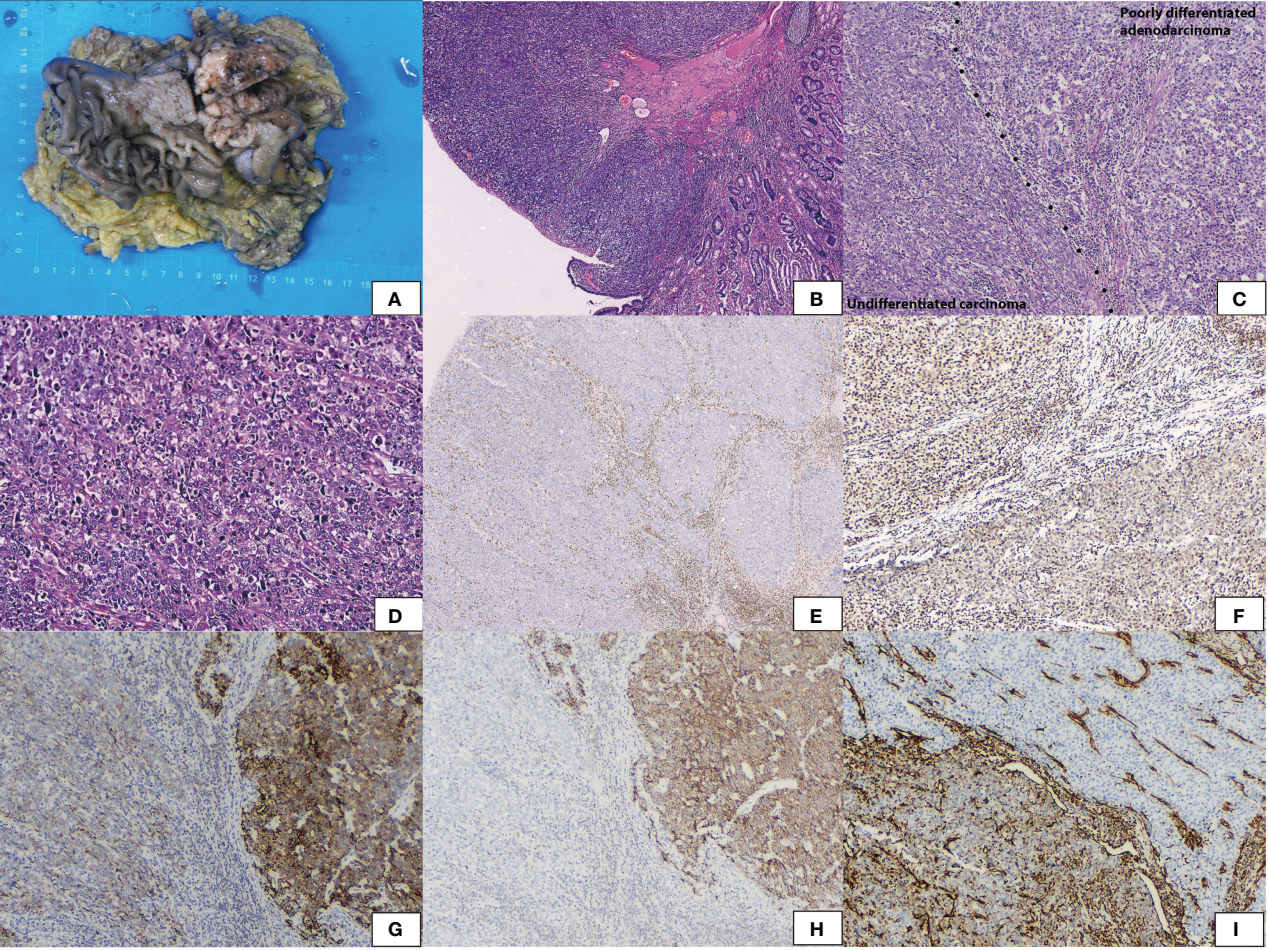
Gross appearance showed **(A)** an elevated solid mass, sized 6.3 cm × 5.1 cm × 1.2 cm in the antrum. **(B)** Hematoxylin and eosin staining showed the tumor abruptly transferred from the normal epithelium. **(C)** The tumor encompassed two components with a clear-cut surface. Poorly differentiated adenocarcinoma with gland formation and undifferentiated carcinoma with diffuse sheets without epithelial differentiation. **(D)** Undifferentiated carcinoma of polygonal cells and pleomorphic giant cells. Rhobdoid cells were common with vesicular nuclei and conspicuous nucleoli. **(E)** Immunohistochemical staining revealed both areas lost SMARCA4. **(F)** SMARCA2 was positive in poorly differentiated adenocarcinoma and was reduced in undifferentiated carcinoma. **(G)** PanCK was positive in poorly differentiated adenocarcinoma and was reduced in undifferentiated carcinoma. **(H)** E-cadherin was positive in poorly differentiated adenocarcinoma and lost in undifferentiated carcinoma. **(I)** Vimentin was positive in undifferentiated carcinoma and lost in poorly differentiated adenocarcinoma.

The pathological findings revealed an elevated solid mass measuring 6.3 cm × 5.1 cm × 1.2 cm (**Figure 2A**) in the antrum next to the pylorus.Microscopically, the tumor invaded the muscularis propria of the gastric wall. It abruptly transitioned from the normal epithelium (**Figure 2B**). Two components were differentiated. One was a poorly differentiated adenocarcinoma with gland formation (**Figure 2C**). The other was an undifferentiated carcinoma with diffuse sheets of polygonal and pleomorphic giant cells. Cells were discohesive. Rhabdoid cells were common; these cells had conspicuous vesicular nucleoli (**Figure 2D**). Both carcinomas lost expression of SMARCA4 (**Figure 2E**) but were positive for SMARCB1, P53, and SALL4. They were negative for CgA, CD56, and CD34. Synaptophysin was weakly positive. The poorly differentiated adenocarcinoma expressed SMARCA2 (**Figure 2F**), panCK (**Figure 2G**), and E-cadherin (**Figure 2H**) but lost vimentin (**Figure 2I**). The undifferentiated carcinoma expressed vimentin but lost E-cadherin and showed reduced expression of panCK and SMARCA2. The tumor was finally diagnosed as SD-DC (T2N0M0).

He did not receive chemotherapy, immune checkpoint inhibitors, or targeted therapy because of his poor physical condition. Follow-up was performed, and the patient was alive after over five months.

### 3.3 Literature review

We retrieved data for “(SMARCA4) AND (gastric carcinoma)” on PubMed and found 29 reported patients described in nine studies (2–10) (**Table 1**), namely, 22 (76%) men and seven (24%) women aged 30–75 years (average: 62.3 years). The neoplasm sites (excluding seven patients with no description) from most to least common were the body (10/22), antrum (7/22), fundus (1/22), cardia (1/22), and angle (1/22); the remaining two involved several sites, including the cardia, fundus, and body. Tumor size ranged from 4 cm to 14 cm (average: 7.3 cm), and nine cases were not described. Metastatic lymph nodes were found in 81% (22/27), and other metastases in 30% (7/23). There were 78% (18/29) stage III or IV cases. The median overall survival was 9 months (2–190.1 months). The histomorphology varied and included diffuse sheets, nests, abortive gland lumens, and tubules of anaplastic epithelioid cell sand scattered rhabdoid multinucleated giant cells. The tumors with sheets were predominant, presenting an undifferentiated pattern in 50% (12/24). Partly glandular or mixed and dedifferentiated carcinoma occurred in 25% (6/24). Tumors with pure nests or diagnosed tubular adenocarcinoma and solid adenocarcinoma occurred in 25% (6/24) (**Table 2**). These tumors were classified according to histomorphology. Although they were not significantly different in clinical characteristics by existing limited data, they had different histomorphology and immunophenotypes. Interestingly, we found SMARCA2 only strongly expressed in adenocarcinoma, irrespective of subtype (**Table 1**). Chang (3) and Huang (4) also performed SMARCA2 staining and found it only in the adenocarcinoma areas but not in undifferentiated carcinoma.

**Table 1:** Clinical characteristics reported in SMARCA4-deficient gastric carcinoma.

No	References	Age/sex	Site	Size (cm)	TNM/metastasis	Treatment	Survival (month)	SMARCA2 expression
1	Agaimy (2)	75/M	Gastric posterior wall	8	Peritoneal and lymph node IV	Resection	NA	Reduced
2	Chang (3)	74/M	Gastric cardia fundus body	8.0	T4N3M1 IV	Resection target therapy	9	–
3		64/M	Gastric angle	NA	T4N3M1 IV	Chemotherapy	3	NA
4		57/M	Gastric antrum	NA	TxNxM1 IV	None	NA	NA
5		58/M	Gastric antrum	NA	T3N2M0 IIIA	Resection chemotherapy	14	NA
6		46/M	Gastric body	NA	TxNxM1 IV	NA	5	+
7		30/M	Gastric body	8	T3N1M0 IIB	Resection chemotherapy	3	–
8	Huang (4)	63/M	Gastric stump	7.5	T3N2M0 IIIA	NA	16.3	Heterogeneous
9		67/F	Gastric body	6.4	T4bN1M0 IIIB	NA	14.2	+
10		76/F	Gastric cardia	4.4	T4aN3bM0 IIIC	NA	4.4	–
11		46/F	Gastric antrum	4.5	T3N1M0 IIB	NA	190.1	–
12		72/M	Gastric antrum	5	T4aN2M0 IIIA	NA	23.7	+
13		62/F	Gastric body	8	T4aN1M0 IIIA	NA	26.4	–
14	Wu (5)	49/M	Gastric body	NA	IV	Resection chemotherapy PD1 immunotherapy target therapy	> 12	NA
15		66/M	Gastric body	NA	IV	Chemotherapy PD1 immunotherapy	> 10	NA
16		64/M	Gastric body antrum	NA	IV	Chemotherapy	2	NA
17		72/F	Gastric body antrum	NA	IV	NA	3	NA
18		72/M	Gastric fundus	NA	NA	NA	Miss	NA
19	Zhang (6)	55/F	Gastric antrum	4	T2N2M0 IIB	NA	11.57	–
20		57/M	Gastric body	8	T4bN0M0 IIIA	NA	8.13	–
21		71/F	Gastric antrum	14	T3N1M0 IIB	NA	5.73	–
22	Chen (7)	76/M	Gastric antrum	9	T4aN3M0 III	Resection chemotherapy	> 18	NA
23	Jin (8)	75/M	Gastric	NA	T4aN2M0 IIIA	Resection	NA	NA
24		75/M	Gastric	NA	T4aN1M0 IIIA	Resection	NA	NA
25		67/M	Gastric	NA	T4bN2M1 IV	NA	NA	NA
26		48/M	Gastric	NA	T4aN2M1 IV	NA	NA	NA
27		51/M	Gastric	NA	T3N0M0 IIA	Resection chemotherapy PD1 immunotherapy	> 7	NA
28	Wang (9)	74/M	Gastric cardia, fundus and body	8	T4aN5M0 III	Resection chemotherapy	8	–
29	Lin (10)	59/M	Gastric body	7	NA	Resection	NA	NA
30	This study	65/M	Gastric cardia	NA	NA	NA	> 3	+
31	This study	75/M	Gastric antrum	6.3	T2N0M0 IIA	Resection	> 5	Reduced

NA, not available; M, male; F, female; +, positive; −, negative.

**Table 2:** Morphological characteristics of reported SMARCA4-deficient gastric carcinoma.

No	Gross	Patterns	Cytology
1	Ulcerated transmural	Partly abortive gland lumens and mucinous differentiation	Anaplastic rhabdoid
2	Giant invasive ulcerated	Big nests and sheets (40%), poor cohesive pseudoglandular (45%), cords (5%), small solid nests (5%), and irregular adenoid pattern without definite lumen formation (5%)	Epithelioid tumor cells Scattered multinucleated giant cells
3	NA	Diffused sheets (> 95%) and cords (< 5%)	Epithelioid tumor cells, with focal clear cell cytoplasm (10%)
4	Ulcerated	Nests (100%)	Epithelioid tumor cells, with focal clear cell cytoplasm (20%)
5	Ulcerated	Diffuse sheets (75%) Poor cohesive nests (25%)	Epithelioid tumor cells Scattered multinucleated giant cells
6	NA	Nests (100%)	Epithelioid tumor cells
7	Endophytic	Undifferentiated component	Rhabdoid tumor cells
8	NA	Tubular adenocarcinoma	NA
9	NA	Tubular adenocarcinoma	NA
10	NA	Mixed carcinoma	NA
11	NA	Solid adenocarcinoma	NA
12	NA	Solid adenocarcinoma	NA
13	NA	Undifferentiated rhabdoid features	NA
14	NA	NA	NA
15	NA	NA	NA
16	NA	NA	NA
17	NA	NA	NA
18	NA	NA	NA
19	NA	Dedifferentiated	NA
20	NA	Dedifferentiated	Undifferentiated cell
21	NA	Undifferentiated	Undifferentiated cell
22	Ulcerative	Undifferentiated	Rhabdoid appearance
23	NA	Diffuse sheet	Anaplastic cell with rhabdoid feature
24	NA	Diffuse sheet	Anaplastic cell with rhabdoid feature
25	NA	Diffuse sheet	Anaplastic cell with rhabdoid feature
26	NA	Diffuse sheet	Anaplastic cell with rhabdoid feature
27	NA	Diffuse sheet +20% glandular	Anaplastic cell with rhabdoid feature
28	Ulcerative	Diffuse sheet	Epithelioid cell with rhabdoid feature
29	Protuberant	Sheet	Round to epithelioid undifferentiated cells
30	Protuberant	Tubular (30%) and nest (70%) with glandular differentiation	Anaplastic epithelioid cell
31	Protuberant	Nest (50%) with glandular differentiation and diffuse sheet (50%)	Anaplastic epithelioid cell and rhabdoid cell

NA, not available.

## 4 Discussion

SMARCA4 is located on chromosome 19p13 and encodes the transcription activator BRG1. It is an ATP-dependent catalytic subunit of SWI/SNF chromatin-remodeling complexes that regulate chromatin structure and gene expression by supplying energy (11). The SWI/SNF chromatin-remodeling complexes usually consist of 12–15 proteins, including ATPase subunits (SMARCA4 and SMARCA2), core subunits (SMARCB1, SMARCC1, and SMARCC2), and various regulatory subunits (ARID1A, ARID1B, and ARID2). The essential diagnostic criteria of SMARCA4-deficient undifferentiated tumor depend on the detection of SMARCA4 (BRG1) deficiency by immunohistochemistry but not a genetic diagnosis (12). Sequencing can be helpful to clarify the significance of reduced expression of SMARCA4, but it is not necessary for the diagnosis, because immunohistochemistry shows complete loss in most cases and is sufficient to document SMARCA4 deficiency. In addition, the mutation may not be detectable, depending on the limitations of the methods used. Because the second hit often copy-neutral loss heterozygosity (i.e., accompanied by duplication of the mutated allele) (13).

SMARCA4 loss is characteristic of thoracic sarcomas but, now, it represents primarily undifferentiated and dedifferentiated carcinomas rather than primary thoracic sarcomas (13). It has been sporadically identified in human carcinomas in a variety of regions, including endometrioid adenocarcinoma, non-small cell lung carcinoma, carcinoma of the sinonasal tract, and small cell carcinoma of the ovary-hypercalcemic type (14). SMARCA4-deficient carcinoma has an extremely low incidence. A literature search on SMARCA4-deficient gastric carcinoma returned 29 cases. Huang et al. screened SMARCA4 alterations using immunohistochemistry on 1,199 surgically resected gastric carcinomas and, in only six (0.5%), SMARCA4 was completely lost (4).

We reported two cases and reviewed the literature to classify these rare tumors. The clinicopathological features of our cases and reported cases were as follows (1): The tumor often occurred in middle-aged and older patients, 30–75 years old (average age: 62.3 years). Males predominated (77% [20/26]), and the clinical stages were III or IV in 78% (18/23). There was rapid progression and poor outcomes; the median overall survival was 8 months (3–190.1 months). The effect of conventional chemotherapy was poor (2). Histomorphologically, the tumors demonstrated sheets, trabecular, solid, nest, abortive gland, tubular distribution, and large epithelioid or rhabdoid cells with low adhesion. Anaplastic cells had vesicular nuclei, prominent nucleoli, and high mitosis indexes (3). All tumors lost SMARCA4 expression; panCK was negative, and SMARCA2 was reduced or lost in the undifferentiated carcinomas. SMARCA2 was expressed in epithelial differentiation. In our cases, differentiated adenocarcinoma expressed SMARCA2, panCK, and E-cadherin but lost vimentin. The undifferentiated carcinoma expressed vimentin and but lost E-cadherin and showed reduced panCK and SMARCA2.

Considering the histomorphology and histochemistry phenotype, we propose a new category: SMARCA4-deficient gastric carcinoma, which may be divided into three subtypes (1): SD-UC, demonstrating diffuse sheets without epithelioid differentiation. Rhabdoid cells occur frequently and may be prominent. There are discohesive cells with anaplastic features. SMRCA2, E-cadherin, and epithelioid markers are negative or reduced, but vimentin is positive (2). SD-DC, demonstrating partly adenoid differentiation in SD-UC, as Chang (3) recommended. The adenocarcinoma areas express epithelioid markers, E-cadherin, and SMARCA2 but are negative for vimentin. The opposite is seen in the undifferentiated areas (3). SD-AD, encompassing purely gland, abortive glands, or nests like conventional adenocarcinoma without rhabdoid or discohesive cells. Epithelioid markers, E-cadherin, and SMARCA2 are positive.

Vimentin and E-cadherin are markers of epithelial-mesenchymal transition expressed in the undifferentiated and glandular areas, respectively, in SD-DC. In our cases, a novel observation in SD-DC was the notable loss of SMARCA2 in the transition from adenocarcinoma to undifferentiated carcinoma. In the reported literature, we also found SMARCA2 expressed in the adenocarcinoma areas in SMARCA4-deficient carcinoma. This finding suggests that SMARCA2 expression changes in the transition to SMARCA4-deficient carcinomas, as Rekhtman proposed (13).

Diagnosing this rare entity is often challenging and relies on an extensive panel of immunohistochemical stains to exclude various morphologic mimics such as neuroendocrine carcinoma, melanoma, and small cell carcinoma of the ovary-hypercalcemic type.

### 4.1 Large cell neuroendocrine carcinoma

Tumors are solid with nests or pseudoglandular epithelioid monoclonal and adhesive cells. It expresses epithelioid markers and at least two neuroendocrine markers. SD-UC may express synaptophysin, mainly focal or weakly positive.

### 4.2 Melanoma

Immunohistochemical detection of HMB45, Melan-A, and S-100 is helpful.

### 4.3 SMARCA4-deficient malignant rhabdoid tumors

These tumors show substantial overlap in histomorphology and immunohistochemistry. Malignant rhabdoid tumors predominantly occur in children under 3 years old.

### 4.4 Metastatic small cell carcinoma of the ovary-hypercalcemic type

This tumor displays unique immune features, including the expression of Wilms’ tumor suppressor gene 1, EMA, vimentin, cytokeratin, and neuroendocrine markers.

SMARCA4-deficient gastric carcinoma is aggressive and resistant to traditional chemotherapy. Based on the antagonism of SWI/SNF and polycomb repressive complex2 (PRC-2), SWI/SNF deletion leads to loss inhibition of enhancer of zeste homolog 2 (EZH2) methyltransferase, which accelerates PRC2-mediated tumorigenesis (15). Urgent, efficient therapy is required. Tazemetostat is a small molecule enhancer of the EZH2 inhibitor approved by the U.S. Food and Drug Administration in 2020 for treating INI1-negative or SMARCA4-negative tumors. Tazemetostat reduces the trimethylation of H3K27 and induces durable tumor responses. Data from a phase I clinical trial of EZH2 inhibitors showed clinical activity consisting of objective responses (complete responses and partial responses) or prolonged stable disease (6.4 to > 20 months), which exceeded 2 years in 5 (38%) of 13 patients with INI1-negative or SMARCA4-negative solid tumors (16).

In conclusion, SMARCA4-deficient gastric carcinoma should be divided into three subtypes: SD-UC, SD-DC, and SD-AD, depending on histomorphology and immunophenotype. Even though they have no significant clinical characteristics, they have different histomorphology and immunohistochemistry. We must recognize these subtypes and collect more cases to characterize SMARCA4-deficient gastric carcinoma.

## Data availability statement

The original contributions presented in the study are included in the article/supplementary material. Further inquiries can be directed to the corresponding authors.

## Ethics statement

The studies involving humans were approved by The Ethics Committee of the Affiliated Zhongshan Hospital, Xiamen University. The studies were conducted in accordance with the local legislation and institutional requirements. The participants provided their written informed consent to participate in this study. Written informed consent was obtained from the individual(s) for the publication of any potentially identifiable images or data included in this article.

## Author contributions

ZYL: Funding acquisition, Writing – original draft, Writing – review & editing. QL: Data curation, Conceptualization, Writing – review & editing, Validation. YH: Data curation, Conceptualization, Writing – review & editing, Investigation, Software. SG: Data curation, Investigation, Conceptualization, Writing – review & editing. YY: Conceptualization, Data curation, Writing – review & editing. ZJL: Validation, Writing – review & editing, Data curation, Supervision, Writing – original draft.
